# Characterization of VOC Emission from Materials in Vehicular Environment at Varied Temperatures: Correlation Development and Validation

**DOI:** 10.1371/journal.pone.0140081

**Published:** 2015-10-09

**Authors:** Jianyin Xiong, Tao Yang, Jianwei Tan, Lan Li, Yunshan Ge

**Affiliations:** 1 School of Mechanical Engineering, Beijing Institute of Technology, Beijing, China; 2 State Key Laboratory of Subtropical Building Science, South China University of Technology, Guangzhou, China; University of Texas Health Science Center at Austin, UNITED STATES

## Abstract

The steady state VOC concentration in automobile cabin is taken as a good indicator to characterize the material emission behaviors and evaluate the vehicular air quality. Most studies in this field focus on experimental investigation while theoretical analysis is lacking. In this paper we firstly develop a simplified physical model to describe the VOC emission from automobile materials, and then derive a theoretical correlation between the steady state cabin VOC concentration (*C*_a_) and temperature (*T*), which indicates that the logarithm of *C*_a_/*T* ^0.75^ is in a linear relationship with 1/*T*. Experiments of chemical emissions in three car cabins at different temperatures (24°C, 29°C, 35°C) were conducted. Eight VOCs specified in the Chinese National Standard GB/T 27630–2011 were taken for analysis. The good agreement between the correlation and experimental results from our tests, as well as the data taken from literature demonstrates the effectiveness of the derived correlation. Further study indicates that the slope and intercept of the correlation follows linear association. With the derived correlation, the steady state cabin VOC concentration different from the test conditions can be conveniently obtained. This study should be helpful for analyzing temperature-dependent emission phenomena in automobiles and predicting associated health risks.

## Introduction

The emission of volatile organic compounds (VOCs) from automobile cabin materials is one of the main causes of poor vehicular air quality [1]. The existence of VOCs can lead to sick car syndrome (SCS) particularly in new cars [2]. In addition, formaldehyde is regarded as a human carcinogen, and benzene may have a carcinogenic effect on the human body, and are thus of particular concern [3–6]. Since people spend long periods of time in automobile cabins for business, shopping, recreation or travel purposes [7], a study of the emission characteristics of VOCs in the vehicular environment is urgently needed, so as to realize effective source control and reduce adverse health risks for the driver and passengers.

Previous studies on these VOC emission characteristics have mainly focused on material emission behaviors in the built environment. In addition, several studies have been performed to measure the pollution level of VOCs in aircraft cabins recently [8, 9]. However, studies targeting material emission in vehicular environment have seldom been reported. VOC emissions from building materials can be characterized by three key parameters: the initial emittable concentration (*C*_m,0_); the material/air partition coefficient (*K*); and the diffusion coefficient inside the material (*D*_m_) [10–21]. If these three parameters are determined, the VOC concentration or emission rate over time in an environmental chamber or indoor environment can be predicted. These key parameters are not only dependent on the physical and chemical properties of the material-VOC combinations, but are also dependent on environmental conditions such as temperature and relative humidity [22, 23]. Huang et al. [24] derived a theoretical correlation between *C*_m,0_ and temperature (*T*) for formaldehyde emission from medium density fiberboard. This correlation shows that the logarithm of *C*_m,0_ *T* ^0.5^ is linearly related to the reciprocal of temperature. In addition, Zhang et al. [25] and Deng et al. [26] established theoretical associations between *K* and *T*, and *D*_m_ and *T*, respectively, showing that *K* decreases with increasing *T* while *D*_m_ increases with increasing *T*. It should be noted that the materials used in the built and vehicular environments are different. The materials used in buildings include many kinds of wood-based boards (e.g., medium density fiberboard, particle board, gypsum board), furniture, wallpaper, marble, while the materials used in automobile cabins consist of plastic, leather, fabric, adhesive. It is still unknown whether the results obtained from tests or analysis of building materials are also suitable for vehicular materials, and further validation is needed.

The steady state VOC concentration in the automobile cabin is generally regarded as a good indicator to evaluate the vehicular air quality and is also convenient for engineering applications, thus is measured by some researchers. If the key parameters of VOC emissions from vehicular materials change with temperature as they do for building materials, the steady state cabin VOC concentration will change with temperature, since this concentration is a function of the three key parameters and reflects the comprehensive effects of these parameters. Some experimental work has been performed to investigate the dependence of the steady state cabin VOC concentration on temperature. Yoshida and Matsunaga [27] reported that the steady state interior concentrations in summer were higher than the concentrations in winter for most of the aliphatic hydrocarbons in the tested automotive cabins. You et al. [28] showed that the concentrations of TVOC and toluene in the three vehicles tested increased sharply when temperature rose from 25°C to 60°C. Chen et al. [29] tested 22 public buses and found that when the temperature increased from 26.9°C to 29.8°C, the concentrations of benzene, toluene, ethylbenzene and xylenes increased by 15.9 μg/m^3^, 73.6 μg/m^3^, 14.2 μg/m^3^ and 37.4 μg/m^3^, respectively. Kim et al. [2] revealed that the steady state emission rate (similar to steady state concentration) of TVOC increased by 11 and 45 fold for two kinds of cabin materials when the temperature increased from 30°C to 90°C. In contrast to the narrow temperature ranges found in indoor environments (e.g., Hunt and Gidman [30] measured the temperature in 1000 buildings in the UK, and observed a maximum temperature of 29°C), the temperature in vehicular environments can reach very high levels, especially on hot summer days due to solar radiation (e.g., Mclaren et al. [31] measured a maximum temperature of 67°C in cars). It is therefore important to study temperature-related VOC emission mechanisms in the vehicular environment. However, the existing studies all focus on experimental investigations, and theoretical analysis is lacking. Without a theoretical correlation, it is impossible to predict the steady state cabin VOC concentration at temperatures other than those used during testing. Therefore, it is necessary to obtain a theoretical association between the steady state cabin VOC concentration and temperature. In addition, the ventilation rate in the automobile cabin changes markedly when a car is moving, which will obviously affect the steady state cabin VOC concentration. Taking this factor into consideration during the theoretical analysis is a challenging problem.

Given the above, the main objective of this paper is to derive a theoretical correlation between the steady state concentration and temperature for VOC emission from materials in vehicular environment. Data from experiments performed on three cars at different temperatures as well as data from literature are used to validate the derived correlation.

### Development of the correlation

For VOC emission from board materials in chamber or real environment, the following assumptions are generally applied to simplify the analysis of the problem, i.e.: (1) the board material is single-layer and homogeneous; (2) the VOC diffusion process inside the board material is one-dimensional; (3) the VOC in the chamber or environment is well mixed. The analysis of assumptions (1) and (2) is included in the “Results and discussion” section. With these assumptions, the VOC concentration in the chamber or environment can be described by [14]:

$$C_{\text{a}}=2C_{\text{m},0}\sum_{n=1}^{\infty }\frac{\beta q_{n}\sin q_{n}}{G_{n}}\exp(-D_{\text{m}}\delta^{-2}q_{n}^{2}t)$$ (1)

where, *C*_a_ is the concentration of VOC in the chamber or environment, μg/m^3^; *C*_m,0_ is the initial emittable concentration of VOC in the material, μg/m^3^; $G_{n}=[K\beta +(\alpha -q_{n}^{2})KBi_{\text{m}}^{-1}+2]q_{n}^{2}\cos q_{n}+[K\beta +(\alpha -3q_{n}^{2})KBi_{\text{m}}^{-1}+\alpha -q_{n}^{2}]q_{n}\sin q_{n}$, *β* = *Aδ* / *V*; *α* = *Qδ* ^2^ / *D*_m_ *V*; *Bi*_m_ = *h*_m_ *δ* / *D*_m_; *D*_m_ is the diffusion coefficient of VOC in the material, m^2^/s; *δ* is the thickness of the material, m; *A* is the emission area of the material, m^2^; *h*_m_ is the convective mass transfer coefficient, m/s; *K* is the material/air partition coefficient; *q*_*n*_ are the positive roots of

$$q_{n}\tan q_{n}=\frac{\alpha -q_{n}^{2}}{K\beta +(\alpha -q_{n}^{2})KBi_{\text{m}}^{-1}}\;(n=1,2,\ldots )$$ (2)

Eq 1 is derived based on VOC emission from board materials in buildings. Although the types of materials used in buildings and automobiles are different, there are still some similarities between them in the emission behaviors. In both cases there are three emission processes: the diffusion process within the material, the partition process at the material/air interface, and the convective mass transfer process along the material surface. We therefore make a rational assumption that Eq 1 is also applicable to characterize the emission scenarios of VOC from materials in vehicular environment. This assumption will be validated in the following section by comparing the derived correlation (based on Eq 1) with experimental data.

Eq 1 is applicable for predicting both the short-term and long-term emission behaviors. The calculation of *C*_a_ with Eq 1 needs to find the roots of transcendental Eq 2 and perform sums on infinite exponential terms, which is relatively complicated for engineering applications. For materials used in vehicular or built environment, the emission period may last for several years, which is much longer than the time to reach steady state (see the analysis in the “Experimental section”). So, the VOC emission is mainly in the long-term emission period, or reaches steady state. For this emission period, Eq 1 can be simplified.

Our previous study [32] reveals that, for the infinite exponential series as in Eq 1, the terms decay very fast. When the Fourier number (*Fo*_m_, defined as *D*_m_ *t*/*δ* ^2^) is over 0.125, the relative error is no more than 5% when applying the first term to approximately substitute the whole terms of the infinite exponential series. For long-term emissions or at steady state, the *Fo*_m_ is larger than approximately 0.2 [33]. Therefore, Eq 1 can be simplified by only keeping the first term (*n* = 1). This means:

$$C_{\text{a}}=2C_{\text{m},0}\beta \frac{q_{1}\sin q_{1}}{G_{1}}\exp(-D_{\text{m}}\delta^{-2}q_{1}^{2}t)$$ (3)

For most material emission scenarios in vehicular and built environments, *Bi*_m_/*K* is in the range of 20–700 [34], *α* is in the range of 60–36200 [33, 35], and *α*/*Kβ* is in the range of 7–2900 (ACH changes in the range of 0.5-2/h) [10, 32, 36]. For VOC emission from board materials under ventilated conditions, the first root *q*_1_ of Eq 2 is in the range of 0-*π*/2. By applying the above parameter ranges to perform dimensional analysis on Eq 2, we get:

$$\alpha -q_{1}^{2}\approx \alpha ,\;\alpha >>K\beta +\alpha KBi_{\text{m}}^{-1}$$ (4)

Eq 4 indicates that the denominator is far more than the numerator in the right hand of Eq 2 for the first root. Therefore, the first root *q*_1_ can be approximately taken as *π*/2. With this root, and considering that *α* is much larger than *Kβ* and *q*_1_, *G*_1_ is approximately equal to *αq*_1_sin*q*_1_. Substitute this into Eq 3, it yields:

$$C_{\text{a}}=2C_{\text{m},0}\frac{\beta }{\alpha }\exp(-D_{\text{m}}\delta^{-2}q_{1}^{2}t)=2C_{\text{m},0}D_{\text{m}}\frac{A}{\delta Q}\exp(-D_{\text{m}}\delta^{-2}q_{1}^{2}t)$$ (5)

This equation can be also rewritten as in the form of *Fo*_m_:

$$C_{\text{a}}=2C_{\text{m},0}D_{\text{m}}\frac{A}{\delta Q}\exp(-2.46Fo_{\text{m}})$$ (6)

Eq 6 is similar to the result derived based on Little et al.’s model [37]. This equation implies that the steady state or long-term VOC concentration in vehicular environment is in an exponential decay relationship with the emission time, which conforms with some empirical models [38]. In addition, Eq 6 indicates that the long-term VOC concentration is just related with the internal material properties (area, thickness, key parameters) and the external ventilation conditions, which is consistent with previous studies based on dimensionless analysis [33, 39]. In these two studies, the long-term emission rate is irrelevant with the chamber volume, which corresponds to that the long-term concentration is also irrelevant with the chamber volume. By extending it to vehicular environment, the long-term concentration should be independent on the vehicle volume, as Eq 6 revealed. Compared with the complicated fully-analytical model (Eq 1), the simplified model (Eq 6) is more convenient for calculation and analysis, thus will be helpful for screening level of steady state or long-term emissions. It should be pointed out that a similar form of Eq 6 can be derived from another angle, which can be regarded as a validation of Eq 6, and is introduced in S1 Text of the Supporting Information.

Eq 6 is derived from physical model, while S1 equation (S4) in the Supporting Information is obtained from semi-empirical correlations. We can see that these two equations are very similar, just a minor difference in the constant. The relative deviation between these two equations in predicting the steady state VOC concentration is about 5%, which is acceptable for engineering applications. The consistency in these two equations validates the effectiveness of the simplification process from Eq 1 to Eq 6.

Since Eq 6 is a mechanistic model and has clear physical meaning, we apply it to analyze the impact of temperature on the emission characteristics. Huang et al. [24] and Deng et al. [26] derived the theoretical correlations between *C*_m,0_ and temperature (*T*), and *D*_m_ and *T*, respectively. These correlations are described by Eqs 7 and 8:

$$C_{\text{m},0}=\frac{C_{1}}{T^{0.5}}\exp(-\frac{C_{2}}{T})$$ (7)

$$D_{\text{m}}=D_{1}T^{5/4}\exp(-\frac{D_{2}}{T})$$ (8)

where, *C*_1_, *C*_2_, *D*_1_, *D*_2_ are positive constants, which are independent of temperature and are only related to the physical and chemical properties of the material-VOC combinations.

Substitute these two correlations into Eq 6, we get:

$$C_{\text{a}}=\frac{E_{1}}{Q}T^{0.75}\exp(-\frac{E_{2}}{T})\exp(-2.46Fo_{\text{m}})$$ (9)

where, *E*_1_ = 2*C*_1_ *D*_1_ *A*/*δ*, *E*_2_ = *C*_2_+*D*_2_.

By taking the logarithm on both sides of Eq 9, it gives:

$$\ln\frac{C_{\text{a}}}{T^{0.75}}=\ln\frac{E_{1}}{Q}-\frac{E_{2}}{T}-2.46Fo_{\text{m}}$$ (10)

The parameter *E*_2_ is in the 10^4^ orders of magnitude [24, 26], so the value of *E*_2_/*T* is about 30 for a normal car cabin temperature range. Given that *Fo*_m_ is larger than approximately 0.2 when the emissions reach steady state [33], the term (-2.46*Fo*_m_) is relatively small compared with other terms in Eq 10, and can thus be ignored. In some cases ignoring this term can lead to a small deviation that can be reduced by adjusting the parameters *E*_1_ and *E*_2_ within a narrow fluctuating range. Based upon the above analysis, Eq 10 can be further reduced to:

$$\ln\frac{C_{\text{a}}}{T^{0.75}}=\ln E_{1}-\frac{E_{2}}{T}-\ln Q$$ (11)

Eq 11 indicates that both the temperature and ventilation rate can influence the steady state cabin VOC concentration. In some emission scenarios, these two factors do not change simultaneously. If only the temperature changes while the ventilation rate remains constant, Eq 11 can be written as:

$$\ln\frac{C_{\text{a}}}{T^{0.75}}=F_{1}-\frac{F_{2}}{T}$$ (12)

where, *F*_1_ = ln*E*_1_-ln*Q*, *F*_2_ = *E*_2_.

This correlation establishes the theoretical association between the steady state cabin VOC concentration and temperature, which indicates that the logarithm of *C*_a_/*T* ^0.75^ is in a linear relationship with the reciprocal of *T*. It is easy to demonstrate that *C*_a_ is an increasing function of *T*. Although the cabin VOC concentration at steady sate changes very slowly with emission time, it will still cause considerable change in *C*_a_ when the emission time differs greatly (e.g., one month or above). Therefore, when using correlation (12) to analyze or calculate *C*_a_ at different temperatures, the emission time corresponding to *C*_a_ (at different temperatures) shouldn’t differ substantially.

**Fig 1:**
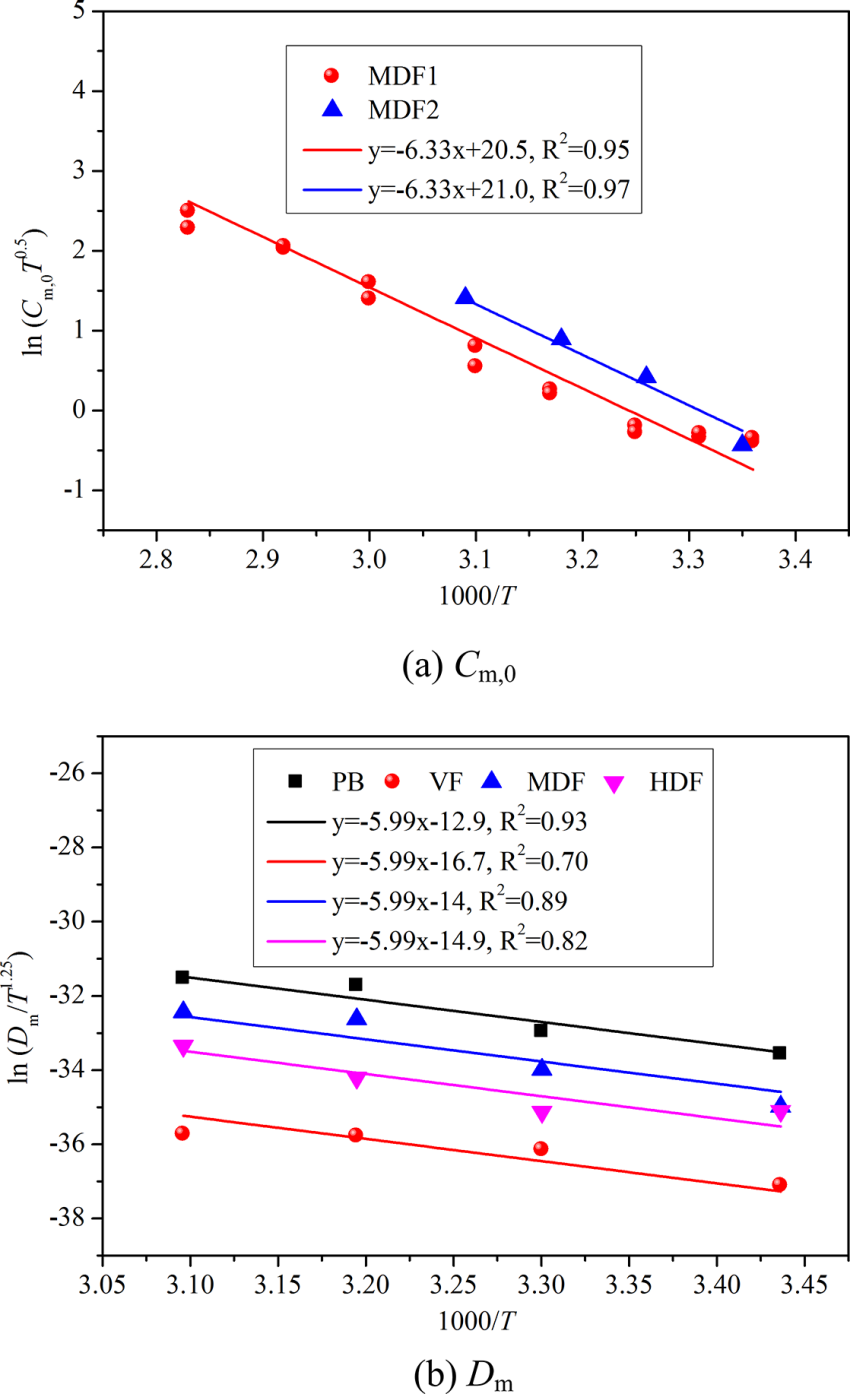
Linear curve fitting results by treating the experimental data with theoretical correlations.

Strictly speaking, the derived correlation (12) is only applicable when VOC is emitted from single-source since the key parameters (*C*_m,0_ and *D*_m_) for different material-VOC combinations may be different. The single-source emission condition can be easily realized in chamber study. However, in real vehicular environment, multi-sources coexist, which makes the temperature-related characteristics complicated. For formaldehyde emission from two board materials (MDF1, MDF2), Huang et al.’s [24] results show that *C*_1_ in Eq 7 is in the range of 1.5×10^8^−7.2×10^9^, and *C*_2_ lies within 6330 ± 552. In addition, for formaldehyde emission from four board materials (PB, VF, MDF, HDF), Deng et al.’s [26] results indicate that *D*_1_ in Eq 8 is in the range of 1×10^−10^–6×10^−3^, and *D*_2_ lies within 5990 ± 1283. This means the parameters *C*_1_ and *D*_1_ change greatly for VOC emission from different sources, while *C*_2_ and *D*_2_ just change in a relatively narrow range. Based on these findings, we can force the *C*_2_ and *D*_2_ for different emission sources as the same (mean value) and re-treat the experimental data, to check whether we can take *C*_2_ and *D*_2_ as unchanged. Fig 1 shows the linear curving fitting results by re-treating the experimental data with theoretical correlations (the logarithm forms of Eqs 7 and 8). The horizontal axis is changed to 1000/*T* to make the figure clearer. This figure indicates that the logarithm of *C*_m,0_ *T* ^0.5^ and *D*_m_/*T* ^1.25^ are in a good relationship with 1/*T* (or 1000/*T*) when we keep the slopes as constants (*C*_2_ = 6330, *D*_2_ = 5990), with most of R^2^ higher than 0.81. Therefore, to perform a preliminary analysis on VOC emission from different sources at various temperatures, we can roughly assume that for a certain VOC emitted from multi-sources, the parameters *C*_2_ and *D*_2_ in Eqs 7 and 8 approximately keep constant, and just *C*_1_ and *D*_1_ vary for different sources. Such treatment is acceptable according to the above analysis, but we acknowledge that future research is needed to further check the feasibility of the assumption.

Under this condition, Eq 9 can be transformed as:

$$C_{\text{a},i}=\frac{E_{1,i}}{Q}T^{0.75}\exp(-\frac{E_{2}}{T})\exp(-2.46Fo_{\text{m}})$$ (13)

where, *C*_a,i_ is the steady state cabin VOC concentration due to the emission of source *i*; *E*_1,i_ = 2*C*_1,i_ *D*_1,i_ *A*_*i*_/*δ*_*i*_, *E*_2_ = *C*_2_+*D*_2_.

It is assumed that the VOC emission from different sources doesn’t interfere with each other. That is to say, the total steady state VOC concentration can be regarded as the accumulation of every source (This will be preliminarily validated in the “Results and discussion” section). By summing on both sides of Eq 13, we get:

$$\sum_{i}C_{\text{a},i}=\frac{\sum_{i}E_{1,i}}{Q}T^{0.75}\exp(-\frac{E_{2}}{T})\exp(-2.46Fo_{\text{m}})$$ (14)

This equation can be rewritten as:

$$C_{\text{a},\text{tot}}=\frac{E_{1,\text{tot}}}{Q}T^{0.75}\exp(-\frac{E_{2}}{T})\exp(-2.46Fo_{\text{m}})$$ (15)

where, *C*_a,tot_ is the total steady state VOC concentration (= $\sum_{i}C_{\text{a},i}$); $E_{1,\text{tot}}=\sum_{i}E_{1,i}$.

By performing similar treatment mentioned above, the following correlation can be obtained:

$$\ln\frac{C_{\text{a},\text{tot}}}{T^{0.75}}=H_{1}-\frac{H_{2}}{T}$$ (16)

where, *H*_1_ = ln*E*_1,tot_-ln*Q*, *H*_2_ = *E*_2_.

Therefore, for VOC emission from multi-sources in vehicular environment, the relationship between the steady state cabin VOC concentration and temperature accords with the same rule as that of VOC emission from single-source. In the following sections, we use experimental data in literature to validate correlation (12), and perform tests in vehicles to validate correlation (16).

### Experimental section

**Fig 2:**
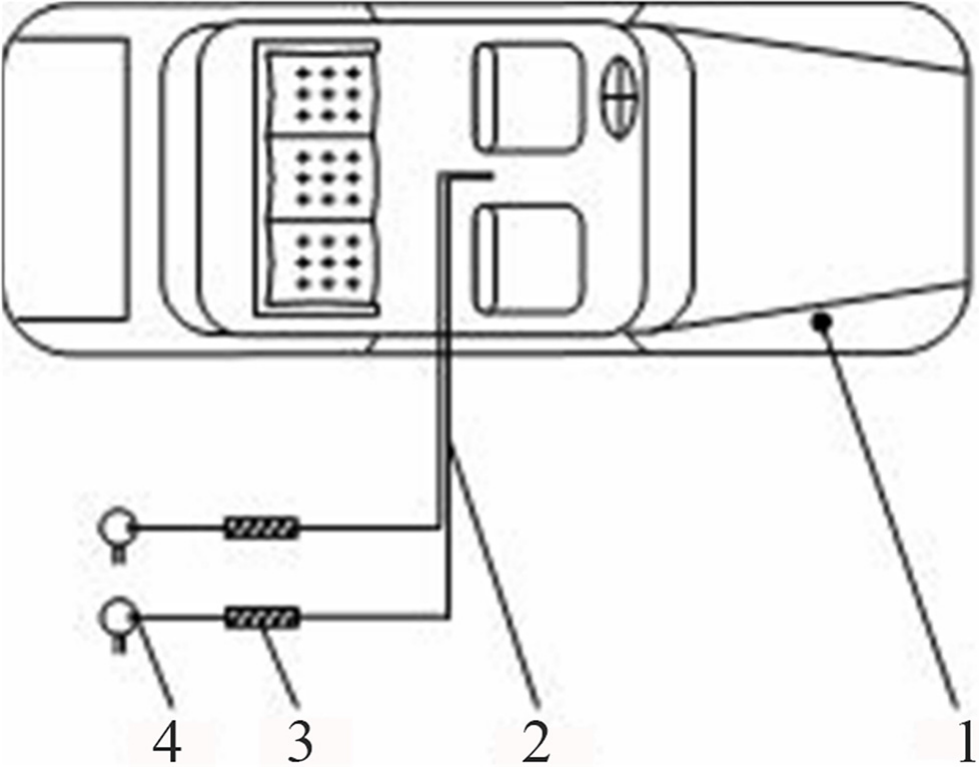
Schematic diagram of the experimental system. (1) tested car; (2) sampling catheter; (3) Tenax-TA tube; (4) sampling pump.

**Fig 3:**
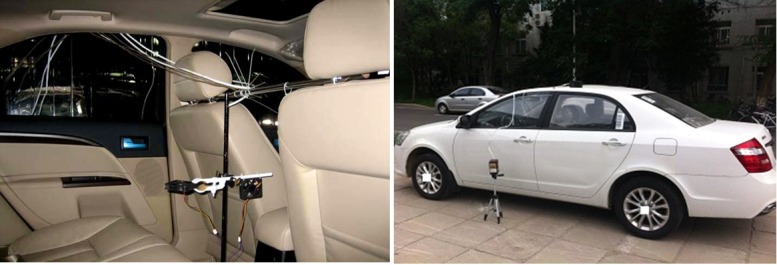
Photo of the experimental system.

Experimental study in three vehicles was conducted to validate the derived correlation on multi-source emissions. The three tested cars were mainstream, mid-range cars (price in the mid-range) manufactured at the same factory. The time since production was less than one month. The experimental system is schematically shown in Fig 2. Fig 3 is a photo from the field tests.

During the experiment, the three tested cars were kept under static conditions, and the windows and doors were closed [40], which is recommended by the Chinese National Standard GB/T 27630–2011 [41]. As pointed out previously, the steady state can be regarded as reached when the *Fo*_m_ (dimensionless emission time) is larger than approximately 0.2. It means that the real emission time should be over 0.2*δ* ^2^/*D*_m_. The materials used in vehicle cabins are generally very thin (e.g., the dashboard, the floor leather), and the thickness can be taken as 0.002m. At present, no study has been performed to measure the *D*_m_ of materials in vehicular environment. As a preliminary estimation, the *D*_m_ of materials in indoor environment is taken for analysis. The median value of *D*_m_ in Xiong et al.’s [32] study is chosen as 3.38 × 10^−10^ m^2^/s. Under this condition, the emission time to reach steady state is calculated to be greater than 0.66h. During the experiment, the three tested cars are maintained under static conditions for 2h, which can ensure the arriving of steady state. In addition, the South Korea Standard [42] also specifies that the tested vehicles should be kept for 2h before sampling the vehicle cabin VOC concentration, which is consistent with the present experimental procedure. The experiments were conducted outdoors. To reduce the impact of outdoor air pollution on the car cabin VOC concentration, a day with good air quality (air quality index, AQI<50) in summer was selected to perform the experiment. To study the impact of temperature on the cabin VOC concentration, the cars were tested at three different temperatures, i.e., 24°C, 29°C, 35°C, which corresponded to typical outdoor summer temperatures for the morning, evening, and noon. The cars were tested in the shadow, and the temperature in the cars was the same as the outdoor temperature. At each temperature, the three cars were tested simultaneously. Since there was no wind during the outdoor environment, the temperature fluctuation was maintained within ±0.5°C during the samplings. The multi-sources that could emit VOC inside the car cabin included plastic, leather, fabric, et al.

In the experiment, eight chemicals specified in the Chinese National Standard GB/T 27630–2011 [41], i.e., benzene, toluene, ethylbenzene, xylene, styrene, formaldehyde, acetaldehyde, acraldehyde, were selected as the target pollutants. The sampling point for the pollutants was set at the intersection of the front seat headrest with the car central axis, and the height of the sampling point approximated to the passenger’s breathing zone (Fig 3). The sampling and analysis of VOCs in the car cabin was primarily based on the EPA (U.S. Environmental Protection Agency) Standard TO-17 [43]. A Tenax-TA tube was used to collect the VOCs from the car cabin, and a secondary thermal desorption-gas chromatography/mass spectrometry (TD-GC/MS) method was applied for analysis. After sampling, the Tenax-TA tube was purged with dry inert gas and then heated. The desorbed VOCs subsequently went into the cold trap with the carrier gas. By performing this secondary thermal desorption, the VOCs finally entered the GC/MS for analysis. The quantitative analysis of VOCs was decided by using the "five-point external standard method". The standard mixed liquid was comprised of benzene, toluene, p-xylene, m-xylene, o-xylene, ethylbenzene, styrene, n-butyl acetate, and n-undecane. For the sampling and analysis of aldehydes and ketones, according to EPA Standard TO-11A [44], a DNPH (dinitrophenylhydrazone)-silica cartridge linked to a pump was used to sample the pollutants in the car cabin. The sampled cartridges were kept in a refrigerator until analysis. After sampling, the DNPH-carbonyl derivatives were extracted with acetonitrile and analyzed by using high performance liquid chromatography (HPLC). For the analysis process, the chromatographic separation of the hydrazones was achieved by use of a C18-column and water/acetonitrile solvent combinations, and an ultraviolet (UV) absorption detector was used for detection.

The sampling flow rate of the pollutants was kept at 100 ml/min, with a sampling time of 30 min. In order to ensure a constant flow, the pump was calibrated by a soap film flowmeter (SKC Inc., U.S.) before and after the sampling process. The flow deviation was less than 5% during the sampling process. Mean value of duplicate samplings were taken as the measured results, for the purpose of reducing the measurement error.

## Results and Discussion

### Validation of correlation (12) with data from literature

**Fig 4:**
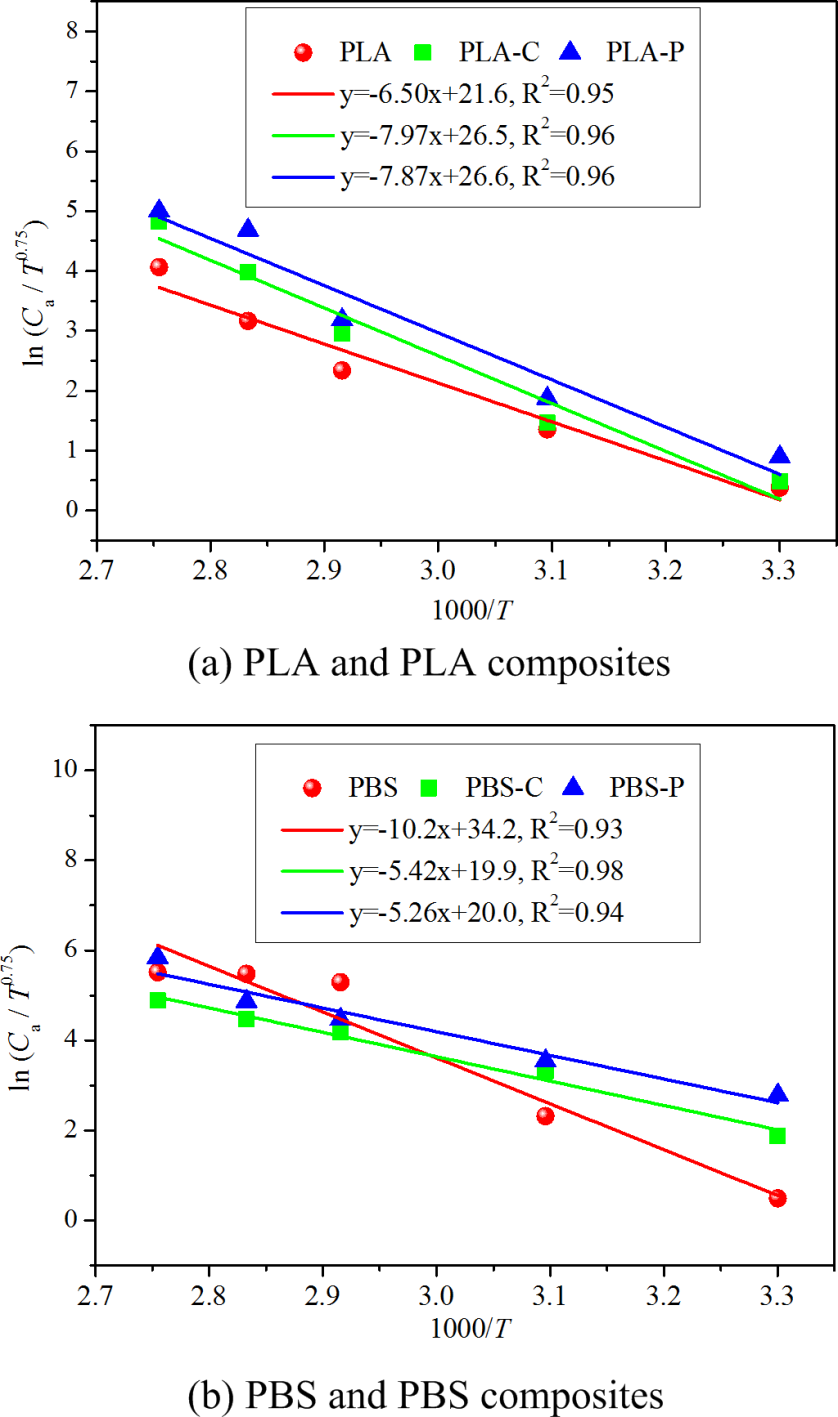
Validation of correlation (12) with Kim et al.’s [2] experimental data from chamber tests.

Correlation (12) is derived based on VOC emission from single-source in vehicular environment. Experimental data found in the literature was used to validate its effectiveness. Kim et al. [2] performed a series of experiments on VOC emission from automobile interior materials in the temperature range of 30–90°C at constant ventilation rate. The interior materials tested include: polylactic acid (PLA), PLA composite with destarched cassava flour (PLA-C), PLA composite with pineapple flour (PLA-P), polybutylene succinate (PBS), PBS composite with destarched cassava flour (PBS-C), PBS composite with pineapple flour (PBS-P). TVOC (total VOCs) was selected for analysis. During these tests, a field and laboratory emission cell (FLEC) was used to obtain the emission rate (the steady state concentration can be calculated based on the emission rate) at various temperatures. Every material was tested in the FLEC individually, which could ensure that the chemical emission was from single-source. The measured experimental data is treated with the derived correlation (12). Fig 4 shows the linear curve fitting results for the six vehicular materials at different temperatures (the horizontal axis is changed to 1000/*T*). According to ASTM Standard D5157-97 [45], R^2^ of 0.81 or greater can be regarded as generally indicative of adequate regression performance. In Fig 4, all the R^2^ values are in the range of 0.93–0.96, implying a high regression accuracy. The good linear association between ln(*C*_a_/*T* ^0.75^) and 1/*T* (or 1000/*T*) demonstrates the effectiveness of correlation (12), as well as the feasibility of extending the application range of Eq 1 from building material emissions to vehicular material emissions.

### Cabin VOC concentrations in the three tested cars

The steady state cabin concentrations of eight VOCs in the three cars were measured during our field tests, and the results are summarized in Table 1. This table shows that the steady state concentrations of all the tested VOCs in the car cabins increase significantly with increasing temperature. The increase tendency is not simply linear, but is greater at high temperatures. As an example we can look at the benzene emission from interior materials in car 2. The cabin benzene concentration at 29°C is about 28.8% higher than that at 24°C (5°C temperature difference). However, the 6°C temperature difference from 29°C to 35°C results in an increase of 102% in the cabin benzene concentration, which is obviously much higher than the increase seen at the lower temperatures. Table 1 indicates that the three pollutants with relatively high concentrations in the tested cars are xylene, formaldehyde and toluene. By comparing the measured concentrations in Table 1 with the upper limit concentration specified for each pollutant in the Chinese National Standard GB/T 27630–2011 [41], we can see that the cabin concentrations of all the VOCs are within the bounds of the standard, with the exception of formaldehyde (100 μg/m^3^) at high temperatures (29°C for car 1, 29°C and 35°C for cars 2 and 3). Since a temperature of 29°C or higher can easily be reached in vehicular environment in summer, the result for formaldehyde indicates that severe adverse health effects to passengers, especially children might emerge. Thus, effective measures should be taken to control and improve the vehicular air quality.

**Table 1:** The measured concentrations of eight VOCs in the three tested cars at different temperatures (unit: μg/m^3^).

VOCs	Car 1			Car 2			Car 3		
	24°C	29°C	35°C	24°C	29°C	35°C	24°C	29°C	35°C
Benzene	4.5	6.5	11.2	8.0	10.3	20.8	4.5	7.7	12.2
Toluene	71.6	85.5	174.2	67.7	96.5	150.7	72.0	95.4	159.4
Ethylbenzene	8.9	10.6	16.8	10.6	11.8	17.7	7.6	9.8	18.0
Xylene	100.5	115.2	165.1	134.5	166.2	203.2	119.1	138.3	199.9
Styrene	3.6	4.8	16.5	3.6	4.2	13.7	5.6	7.3	12.7
Formaldehyde	62.8	64.0	222.9	86.6	100.6	172.8	111.1	115.7	251.6
Acetaldehyde	25.7	28.9	57.0	13.7	18.8	23.1	23.3	27.1	53.6
Acraldehyde	4.5	5.9	6.8	3.4	4.8	7.7	4.5	7.5	14.2

### Validation of correlation (16) with data in the three tested cars

**Fig 5:**
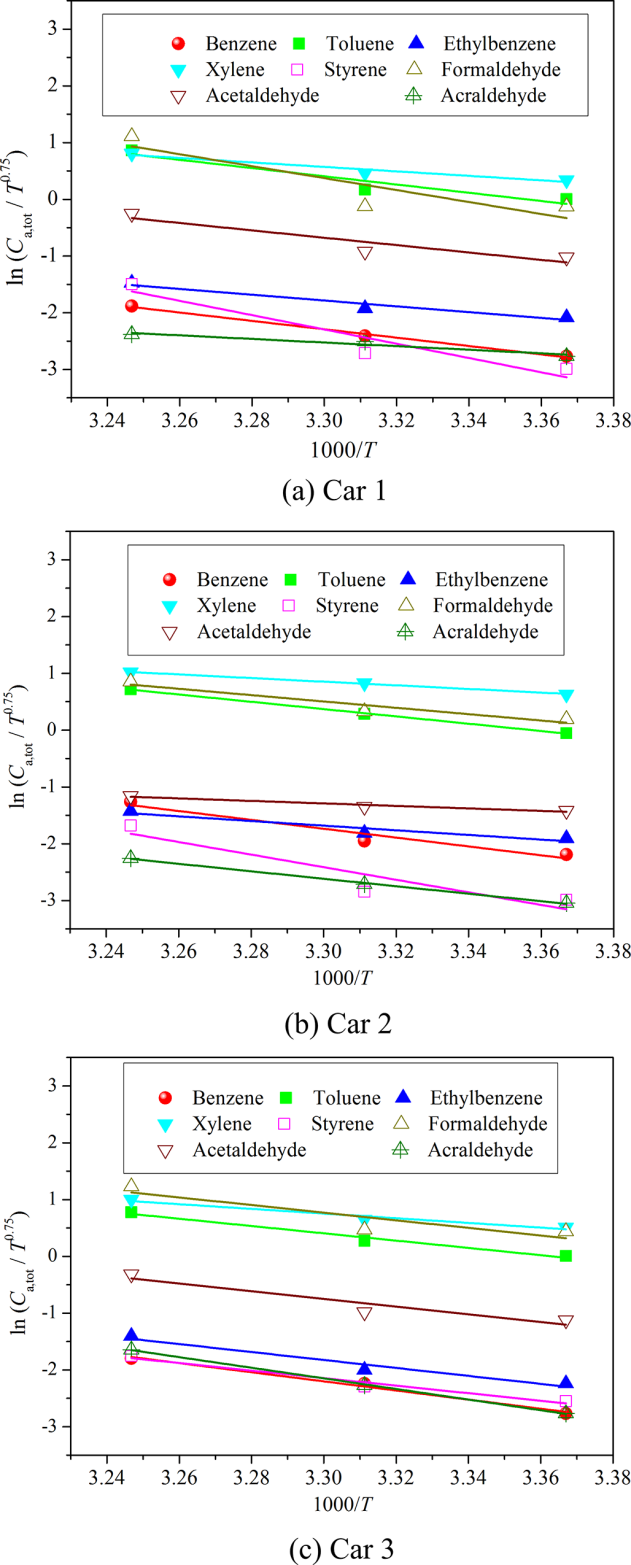
Validation of correlation (16) with experimental data from the three tested cars.

Correlation (16) is applicable when VOC is emitted from multi-sources in vehicular environment. Since the tested three cars include many kinds of emission sources, the measured steady state cabin VOC concentrations at different temperatures can be used to validate the derived correlation. Fig 5 shows the linear curve fitting results for the eight VOCs in the three cars by virtue of correlation (16). The detailed fitting information is summarized in Table 2. Fig 5 as well as Table 2 reveals that the logarithm of *C*_a,tot_/*T* ^0.75^ is in a good linear relationship with 1/*T*, with R^2^ for most of VOCs in the three cars being larger than 0.81, thus validating the effectiveness of the derived correlation.

**Table 2:** Linear curve fitting results using the derived correlation to treat the experimental data in the three tested cars.

VOCs	Car 1			Car 2			Car 3		
	*H*_2_	*H*_1_	R^2^	*H*_2_	*H*_1_	R^2^	*H*_2_	*H*_1_	R^2^
Benzene	7.38 × 10^3^	22.1	0.99	7.80 × 10^3^	24.0	0.90	8.04 × 10^3^	24.3	0.98
Toluene	7.26 × 10^3^	24.4	0.83	6.43 × 10^3^	21.6	1.00	6.42 × 10^3^	21.6	0.97
Ethylbenzene	5.10 × 10^3^	15.1	0.90	4.09 × 10^3^	11.8	0.83	7.00 × 10^3^	21.3	0.92
Xylene	3.94 × 10^3^	13.6	0.90	3.20 × 10^3^	11.4	0.99	4.12 × 10^3^	14.3	0.91
Styrene	12.6 × 10^3^	39.3	0.82	11.1 × 10^3^	34.1	0.72	6.63 × 10^3^	19.7	0.95
Formaldehyde	10.5 × 10^3^	35.1	0.58	5.59 × 10^3^	18.9	0.84	6.71 × 10^3^	22.9	0.62
Acetaldehyde	6.50 × 10^3^	20.8	0.76	2.20 × 10^3^	5.96	0.91	6.80 × 10^3^	21.7	0.81
Acraldehyde	3.17 × 10^3^	7.95	0.89	6.58 × 10^3^	19.1	1.00	9.34 × 10^3^	28.7	1.00

When the ventilation rate keeps constant, the derived correlation (16) involves only two unknown parameters, i.e., *H*_1_ (or ln*E*_1,tot_-ln*Q*) and *H*_2_. Generally, two sets of experimental data relating VOC concentration and temperature are enough to determine these two parameters by solving two equations. If more sets of tests are performed, the two parameters can be obtained by linear curve fitting, which will increase the accuracy of the parameters. Once the two parameters are determined, the correlation can be used to evaluate the VOC concentration at temperatures other than those of the test conditions in vehicular environment, facilitating scientific research as well as engineering applications.

According to the fitting results in Table 2, for benzene emission from interior materials in car 2, the quantitative association between the steady state cabin concentration and temperature can be expressed as:

$$\ln\frac{C_{\text{a},\text{tot}}}{T^{0.75}}=24.0-\frac{7800}{T}$$ (17)

Based on this correlation, the cabin benzene concentration at 70°C can be predicted. The result of 281 μg/m^3^ is about 3 times of the benzene limit specified in the Chinese National Standard GB/T 27630–2011 (110 μg/m^3^). This means that the increase of cabin temperature will greatly increase the health risk and exposure level of driver and passengers in vehicular environment.

### Validation of correlation (16) with data from literature

**Fig 6:**
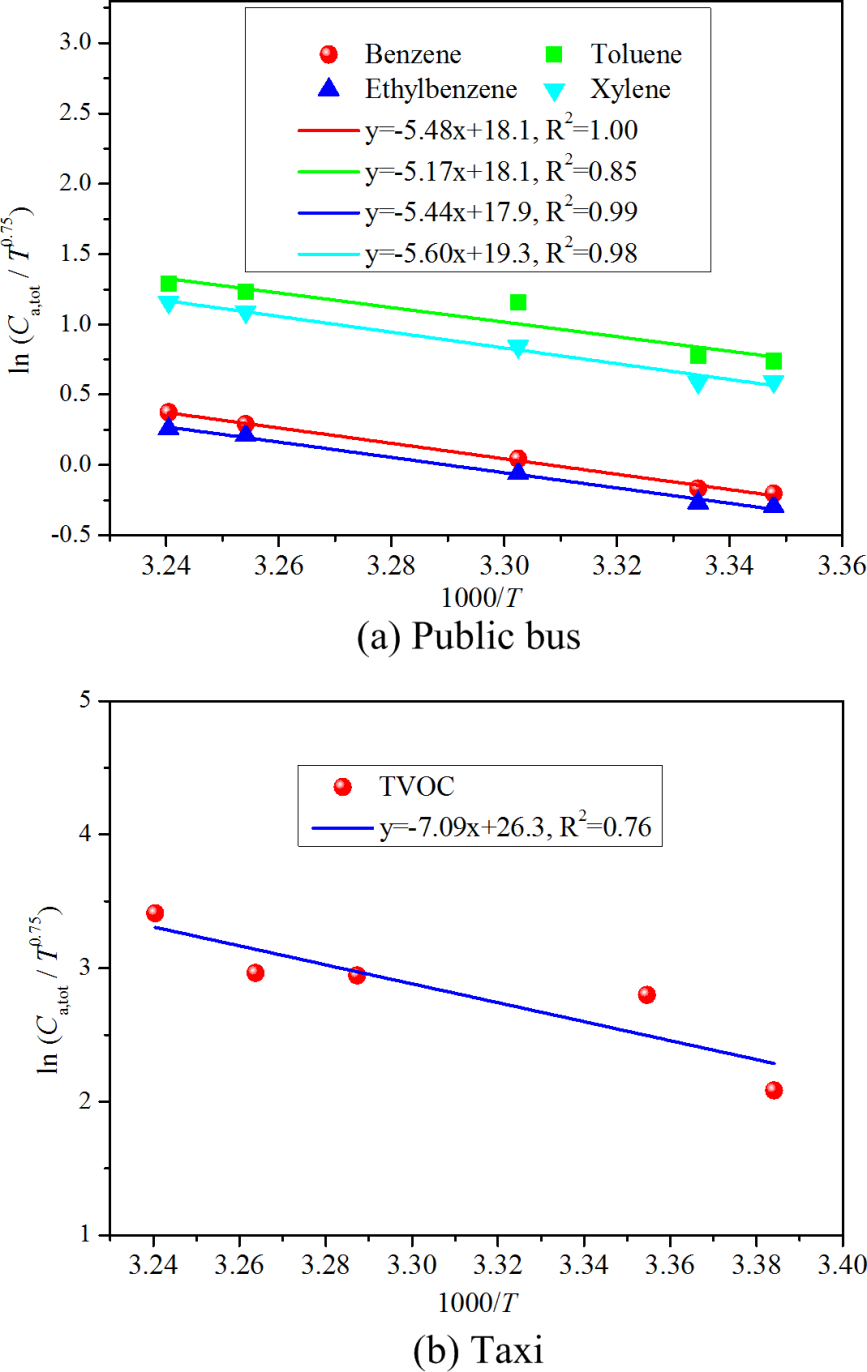
Validation of correlation (16) with Chen et al.’s [29, 46] experimental data from vehicular environments.

**Fig 7:**
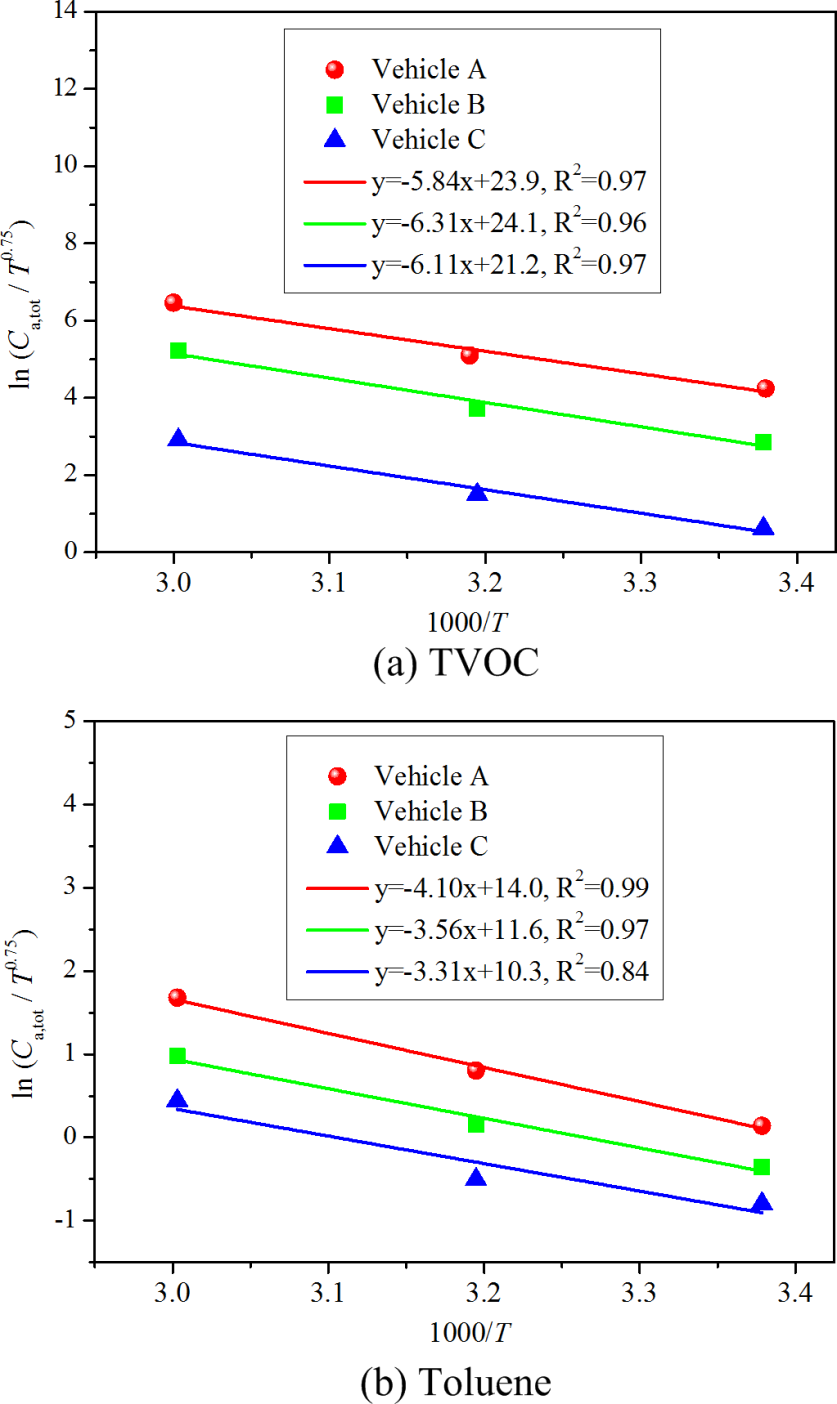
Validation of correlation (16) with You et al.’s [28] experimental data from vehicular environments.

Experimental data in literature is further used to validate the reliability of correlation (16). Chen et al. [29] investigated the VOC concentrations in the cabins of 22 public buses in the temperature range of 25–36°C. The target pollutants included benzene, toluene, ethylbenzene and xylene. The mean value of the measured VOC concentrations inside the cabins of the 22 buses is taken to be the steady state concentration for each pollutant. Fig 6(A) shows the linear curve fitting results of treating the experimental data of cabin concentrations at various temperatures with the derived correlation (16). The figure reveals that, for the four VOCs, the logarithm of *C*_a,tot_/*T* ^0.75^ correlates linearly well with 1/*T*, with the R^2^ values being in the 0.86–0.99 range, thus demonstrating the effectiveness of the derived correlation. This figure further indicates that the slopes of the regression lines are very similar for the four VOCs, meaning that the increasing tendency and level are similar for these pollutants. In addition, Chen et al. [46] also measured the steady state TVOC concentrations in 38 taxi cabin environments from 22°C to 36°C. Fig 6(B) shows the regression results by virtue of the correlation (20). The good linear tendency between the logarithm of *C*_a,tot_/*T* ^0.75^ and 1/*T* illustrates the reliability of the proposed correlation. Validation of correlation (16) with experimental data in other literature (You et al. [28]) is represented in Fig 7.

### Relationship between *H*_2_ and *H*_1_ in correlation (16)

**Fig 8:**
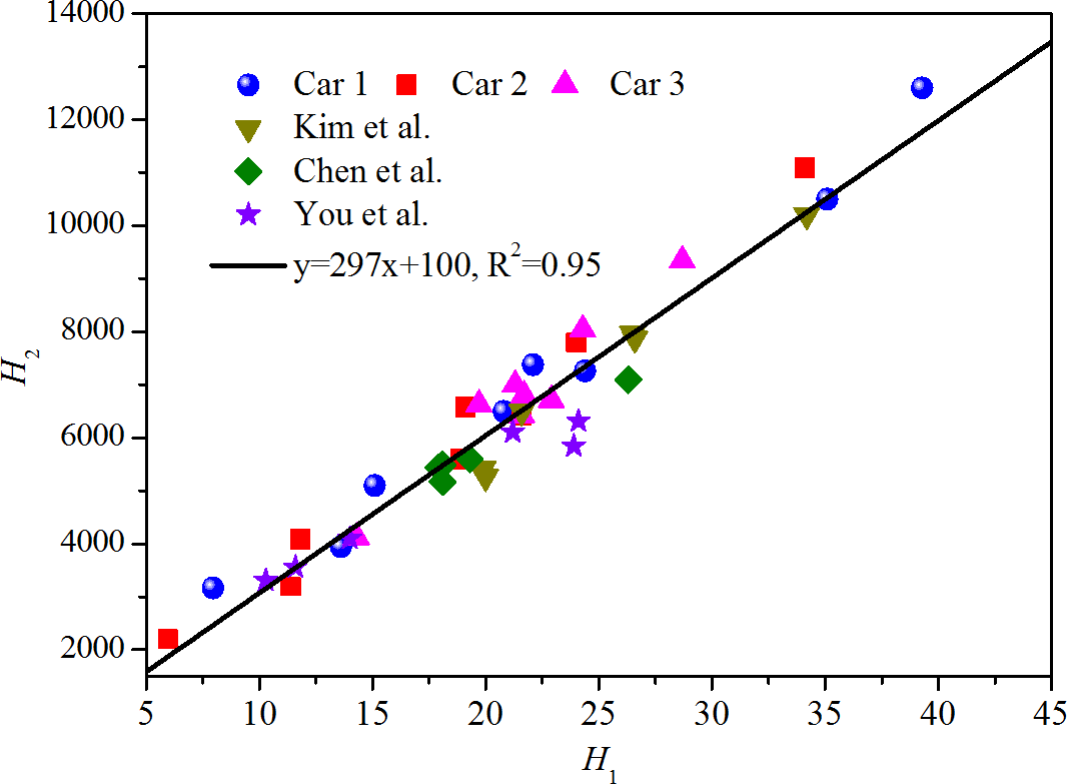
Relationship between *H*_2_ and *H*_1_.

The two parameters *H*_2_ (or *F*_2_) and *H*_1_ (or *F*_1_) in correlation (16) (or correlation (12)) are associated with the physical and chemical properties of material-VOC combinations, such as the adsorption energy, the molar volume of compounds, the maximum adsorbate quantity, etc. These properties may correlate with each other, meaning that *H*_2_ may correlate with *H*_1_. Fig 8 shows the relationship between *H*_2_ and *H*_1_ determined in previous sections by treating the experimental data. This figure reveals that for all the emission scenarios of VOC from vehicular materials in the present study, *H*_2_ is in a good linear relationship with *H*_1_, with the slope of 297 and intercept of 100. Generally, *H*_2_ can be expressed as the ratio of adsorption energy to universal gas constant. The quantitative correlation between *H*_2_ and *H*_1_ provides a pathway to estimate the adsorption energy with some available physical parameters (i.e., the molar volume, the maximum adsorbate quantity). The physical mechanism behind this phenomenon is unknown, and future investigation is needed.

### Analysis on the multi-source emissions

Previously, we made an assumption that the VOC emission from different sources doesn’t interfere with each other (or, the total steady state VOC concentration can be regarded as the accumulation of every source). To validate the rationality of such assumption, a preliminary analysis based on analytical model is performed here. To simplify the analysis of the problem, two source materials are chosen, and the parameters for simulation are set as: *V* = 30L; ACH = 1/h; *A*_1_ = 0.1m×0.1m; *δ*_1_ = 0.0018m; *C*_m1_ = 4×10^7^μg/m^3^; *D*_m1_ = 2×10^-10^m^2^/s; *K*_1_ = 1×10^3^; *h*_m1_ = 2.5×10^-3^m/s; *A*_2_ = 0.1m×0.2m; *δ*_2_ = 0.002m; *C*_m2_ = 1×10^7^μg/m^3^; *D*_m2_ = 1×10^-10^m^2^/s; *K*_2_ = 2×10^3^; *h*_m2_ = 2.5×10^-3^m/s (subscripts 1 and 2 stand for the parameters for materials 1 and 2, respectively). The key parameters *C*_m,0_, *D*_m_ and *K* for the two materials differ by 4 times, 2 times and 2 times, respectively. Xu and Zhang [13] developed an analytical model for VOC emission from single-source. This model can be improved to predict the VOC emission from multi-sources by adding a series of emission or sorption terms in the mass conservation equation in the gas phase. For two-source emission, the mass conservation equation can be improved as:

$$V\frac{dC_{\text{a}}}{dt}=A_{1}\cdot E_{1}+A_{2}\cdot E_{2}-Q\cdot C_{\text{a}}$$ (18)

where, *E*_1_ and *E*_2_ are the emission rates of VOC from materials 1 and 2, respectively.

**Fig 9:**
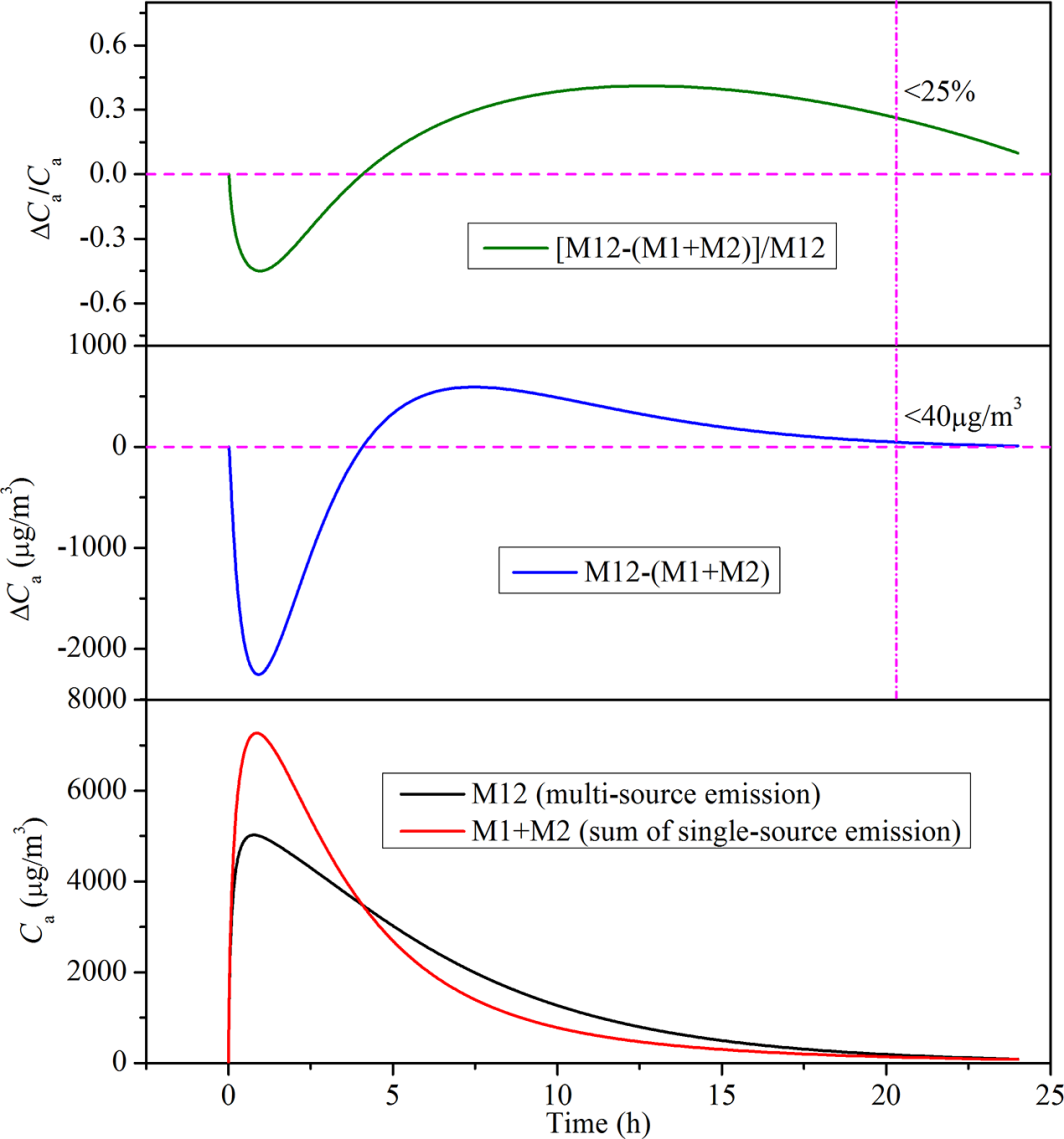
Comparison of chamber VOC concentration between two-source emission and single-source emission.

Based on the improved model, the chamber VOC concentrations from two-source emission and single-source emission can be predicted, and the results are shown in Fig 9. In this figure, M1 stands for the emission when just material 1 is put inside the chamber; M2 stands for the emission when just material 2 is put inside the chamber; M12 stands for the emission when materials 1 and 2 are simultaneously put inside the chamber. Fig 9 reveals that there are some differences in chamber VOC concentration between the two-source emission (M12) and the sum of single-source emission (M1+M2) during the initial and intermediate stage. Before a certain emission time, the chamber VOC concentration of M12 is lower than that of M1+M2. After that time, the change tendency reverses, and the relative deviation between M12 and M1+M2 firstly increases and them decreases to a small value (e.g., 25%) with the increase of emission time. That is to say, for the short-term emissions, the two-source (or multi-source) emission cannot be regarded as the sum of single-source emission for the cases studied; but for the long-term emissions, the two-source (or multi-source) emission can be treated as equivalent to the sum of single-source emission. Such treatment simplifies the analysis of multi-source emission problems while still keeping acceptable accuracy. It should be noted that the present analysis just focuses on two typical materials, further systematic study on more material emission scenarios is still required, to quantitatively determine the equivalent condition (e.g., the range of emission time and key parameters) of multi-source emission.

### Analysis on the multi-layer emissions

The derivation of correlation in the present study is based on the analytical model (Eq 1), which focuses on VOC emissions from single-layer materials. However, some materials used in vehicular environment may be multi-layer, e.g., the seat, thus should be further analyzed. Yao et al. [47] studied the equivalent problem of multi-layer material emissions into single-layer material emissions. A group of two-layer materials with varied key parameter ranges [*D*_m2_/*D*_m1_(0.03), *K*_2_/*K*_1_(2,10)] were simulated. This range can cover the common emission scenarios. The results indicate that when the outer layer is very thin, the two-layer material emissions have equivalent key parameters for most of the simulated parameter ranges. That is to say, The two-layer (or multi-layer) material emissions can be equivalent to single-layer material emissions, by predicting the emission characteristics with single-layer analytical model, together with the equivalent key parameters *C*_m,0_, *K* and *D*_m_. For seats used in vehicular environment, the outer layer is very thin; for other materials used in vehicular environment and accounting for large exposed areas, e.g., the dashboard, the floor leather, they are very thin and are single-layer materials. Given that, to simplify the analysis of the problem, we make a rational assumption of single-layer emissions. The assumption of one-dimensional emission is based on the consideration that the thickness of most of the materials exposed to air in vehicular environment is much thinner than the length and width of the materials.

### Association between ventilation rate and steady state cabin concentration

Besides temperature, ventilation rate (*Q*) is another important factor that will affect the steady state VOC concentration in real automobile cabin especially when the automobile is moving with varied velocity, thus further analysis is needed. When the ventilation rate of automobile changes, things become a little more complicated. It should be noted that an increase in *Q* will not only bring VOCs out of the automobile cabin more rapidly (ventilation dilution), but will also enhance the convective mass transfer coefficient along the material surface (ventilation-mediated convection), which will further influence the VOC concentration in the vehicular environment. Eq 11 in “Development of the correlation” Section just considers the ventilation dilution effect. To take into account the additional ventilation-mediated convection effect, a coefficient (*E*_3_) is introduced, and Eq 11 is modified to be:

$$\ln\frac{C_{\text{a}}}{T^{0.75}}=\ln E_{1}-\frac{E_{2}}{T}-E_{3}\ln Q$$ (19)

When the temperature remains constant, this equation can be rewritten as:

$$\ln\frac{C_{\text{a}}}{T^{0.75}}=J_{1}-J_{2}\ln Q$$ (20)

where, *J*_1_ = ln*E*_1_- *E*_2_/*T*; *J*_2_ = *E*_3_.

This correlation indicates that the logarithm of *C*_a_/*T* ^0.75^ is in a linear relationship with the logarithm of *Q*. It is easy to see that *C*_a_ is a decreasing function of *Q*. For VOC emission from multi-sources in vehicular environment, the *C*_a_ in correlation (20) should be replaced by *C*_a,tot_.

**Fig 10:**
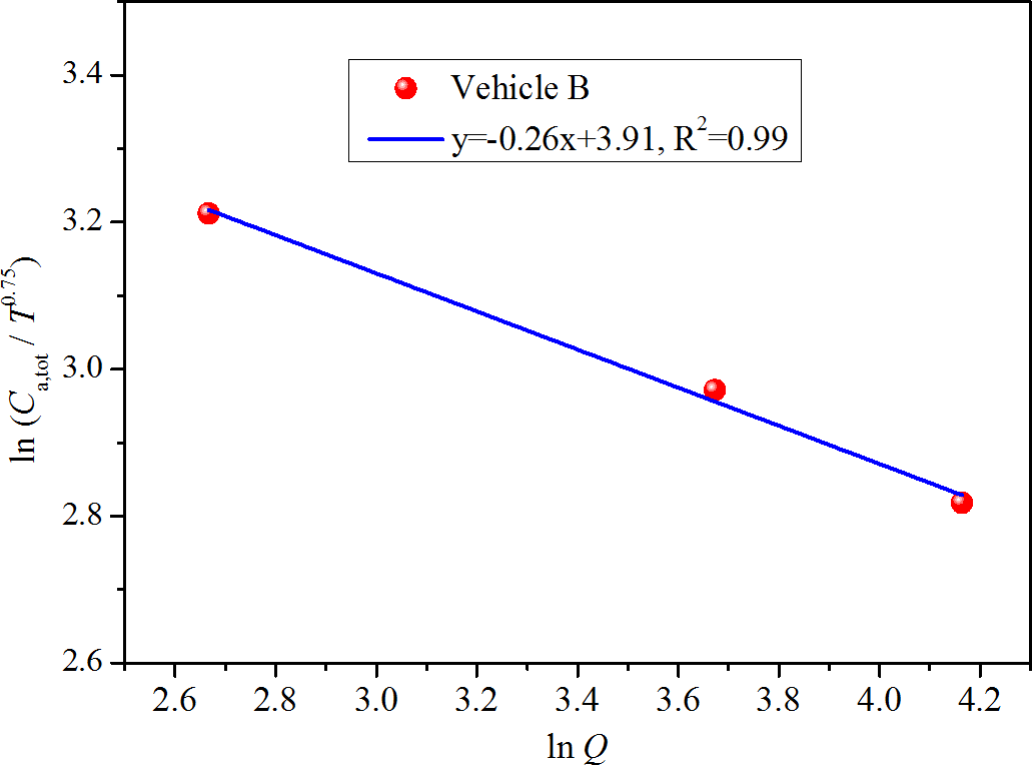
Validation of the correlation (20) with You et al.’s [28] experimental data from vehicular environments.

You et al. [28] tested the changes of steady state chemical concentrations in three vehicles in a room-size environmental chamber (volume: 96 m^3^) under various ventilation rates at the temperature of 25°C. The experimental data is used for validation. During the tests, the change in the steady state TVOC concentration in vehicle B was measured by changing the ventilation rate from 14.4 m^3^/h (0.15 ACH) to 64.3 m^3^/h (0.67 ACH). Fig 10 shows the regression results based on the derived correlation (20) when keeping temperature constant. This figure indicates good linear association between the logarithm of *C*_a,tot_/*T* ^0.75^ and the logarithm of *Q*, thus verifying the effectiveness of correlation (20).

Generally speaking, the use of vehicle is usually accompanied by the change of vehicular environment, e.g., taking a car out from an underground parking lot, opening/closing the air conditioning system or windows when driving. These actions or changes will disturb the original steady state condition, which will cause the changes of the key parameters of VOC emission from vehicular materials, and thus will form a new emission scenario. As discussed previously, it will take the dimensionless emission time of 0.2 (*Fo*_m_, or real emission time of 0.66h) to reach a new steady state. The VOC emissions in vehicular environment are often comprised of a series of different steady states, and the change of environmental conditions will change the emissions from one steady state to another steady state (after taking some time).

It should be noted that the emission characteristics of VOC from materials in real vehicular environment is relatively complicated. In addition to temperature and ventilation rate, the steady state cabin VOC concentration can be influenced by other factors, e.g., relative humidity, solar radiation, time of usage. These impact factors may simultaneously affect the emission characteristics, making the analysis based on physical mechanism complex. This manuscript just considers the impact of temperature and ventilation rate by virtue of physical model, it is therefore can be just regarded as a preliminary study on the material emission characterization in vehicular environment. In addition, the derivation of correlation (16) for multi-source emissions introduces some assumptions, which needs more experimental validation and deep mechanistic analysis. Therefore, future study on this topic is still needed. Considering the existing researches on vehicular environment are all based on experimental investigation, the obtained correlation in the present study provides a theoretical approach, which will be helpful for analyzing the emission phenomena in automobiles under varied environmental factors and predicting the associated health risks.

## Conclusions

This study theoretically and experimentally investigates the impact of temperature on the emission characteristics of VOCs from materials in vehicular environment. A simplified model describing the VOC emission from automobile interior materials at steady state is firstly derived, and then a theoretical correlation between the steady state cabin VOC concentration and temperature is established. The proposed correlation can be applicable for VOC emission from single-source and multi-sources. The effectiveness of the correlation is preliminarily validated by virtue of tests in three car cabins at various temperatures for eight pollutants specified in the Chinese National Standard GB/T 27630–2011, and is further validated by data from the literature. Once the parameters in the derived correlation are determined by two or more sets of experimental data, the correlation can then be applied to predict VOC concentrations when conditions differ from the test scenarios, which is very helpful for engineering applications. In addition, the association between the steady state cabin VOC concentration and ventilation rate is established, which is validated by data in literature. The present study based on theoretical analysis is more advantageous than traditional studies based solely on experimental investigation. Future study will consider the influence of relative humidity and other environmental factors on the emission characteristics of VOCs in the vehicular environment.

## Supporting Information

S1 Text Derivation of Eq 6 from another angle. (DOC)
