# Supplementary material for: Characterization of VOC Emission from Materials in Vehicular Environment at Varied Temperatures: Correlation Development and Validation
Source: PLoS One. 2015 Oct 9;10(10):e0140081. doi: 10.1371/journal.pone.0140081 (PMC4599889; doi:10.1371/journal.pone.0140081)
Supplement: S1 Text — (DOC) [file pone.0140081.s001.doc]

**Supporting Information**

**Manuscript title:**

Characterization of VOC emission from materials in vehicular environment at varied temperatures: correlation development and validation

**Author lists:**

Jianyin Xiong^1,2^, Tao Yang^1^, Jianwei Tan^1,*^, Lan Li^1^, Yunshan Ge^1^

**S1 Text:**

For VOC emission from board materials, Qian et al. [33] obtained a series of correlations between the emission rate and *Fo*_m_, and the steady state emission rate (*E*) can be expressed as [35]:

| $E=2.1\frac{D_{\text{m}}C_{\text{m},0}}{\delta}\exp(-2.36\frac{D_{\text{m}}t}{\delta^{2}})$ | (S1) |
| --- | --- |

VOC mass conservation in the vehicular environment gives:

| $V\frac{dC_{\text{a}}}{dt}=A\cdot E-Q\cdot C_{\text{a}}$ | (S2) |
| --- | --- |

By combining equations (S1) and (S2), and performing integration, the steady state VOC concentration in the vehicular environment is derived to be:

| $C_{\text{a}}=2.1\frac{C_{\text{m},0}D_{\text{m}}A}{\delta V}\frac{1}{\frac{Q}{V}-S}[\exp(-S\cdot t)-\exp(-\frac{Q}{V}\cdot t)]$ | (S3) |
| --- | --- |

where, *S*=2.36*D*_m_/*δ*^2^.

For VOC emission from vehicular and building materials, *Q*/*V* is generally in the order of 10^-4^-10^-3^ s^-1^, while *S* is often in the order of 10^-8^-10^-6^ s^-1^. This means that *S* is much smaller than *Q*/*V*, and accordingly exp(-*S*^.^*t*) is much larger than exp(-*Q*^.^*t*/*V*). Considering that, equation (S3) can be simplified:

| $C_{\text{a}}=2.1\frac{C_{\text{m},0}D_{\text{m}}A}{\delta V}\frac{V}{Q}\exp(-S\cdot t)=2.1C_{\text{m},0}D_{\text{m}}\frac{A}{\delta Q}\exp(-2.36Fo_{\text{m}})$ | (S4) |
| --- | --- |

Equation (S4) is equivalent to the result that takes the term in the left hand of equation (S2), *VdC*_a_/*dt*, as zero, demonstrating that the VOC concentration in vehicular environment changes very slowly when steady state is reached.
